# Dynamic thumb localization and its adaptation: quantification with a novel robotic task

**DOI:** 10.1007/s00221-025-07211-8

**Published:** 2026-01-11

**Authors:** Luis Garcia-Fernandez, Andria J. Farrens, Christopher A. Johnson, Vicky Chan, Joel C. Perry, Eric T. Wolbrecht, David J. Reinkensmeyer

**Affiliations:** 1 Mechanical and Aerospace Engineering Department, University of California Irvine, Irvine, CA 92617 USA; 2 Department of Orthopaedic Surgery and Rehabilitation, division of Physical Therapy, University of New Mexico, Albuquerque, NM 87106 USA; 3 Mechanical Engineering Department, University of Idaho, Moscow, ID 83844 USA

**Keywords:** Thumb proprioception, Stroke rehabilitation, Sensorimotor assessment, Robotic evaluation, Hand function, Proprioceptive adaptation

## Abstract

The thumb plays a crucial role in hand function, yet its proprioceptive abilities remain poorly understood. Here we quantified dynamic thumb localization ability, as well as how this ability adapts to a perturbation, in unimpaired participants. For this, we developed a novel task in which a robot moved the thumb in a circle and participants pressed a button when they felt their thumb aligning with a target point on a screen, receiving visual error feedback in the form of a ball jumping toward the target after they pushed the button. The task also incorporated a propriovisual rotational perturbation to elicit and measure adaptation. To characterize thumb localization ability, we varied thumb speed and rotation diameter, assessed the effect of the propriovisual rotational perturbation, and compared index finger performance. Following task familiarization, average thumb localization error was relatively consistent, with a constant error (CE) of − 5.9°, variable error (VE) of 25.2°, and absolute error (AE) of 29.2°. Errors did not change significantly with speed or circle diameter. Reversing thumb rotation temporarily increased error followed by rapid error adaptation across the next 20 trials, as would be expected if individuals adapted using a body-centered (movement-aligned) frame of reference rather than a world-centered spatial frame. Localization error was comparable for the thumb and the index finger error for the same task and was correlated with a different, robotic assessment of finger proprioception (ρ = 0.61, *p* = 0.001). These findings indicate that dynamic thumb localization is somewhat inaccurate, although it can leverage visual feedback within a body-centered reference frame to adapt. Further, in unimpaired adults, the dynamic localization abilities of the thumb and index finger are related.

## Introduction

For humans, the opposable thumb plays a distinct role in manual dexterity, producing the strongest forces of any of the fingers and being critical for many hand functions. The thumb enables the application of precise grasping and pinching forces, which are essential for object manipulation. Surgeons estimate the thumb contributes to at least 40% of overall hand functionality (Verdan 1968; Doege and Houston 1993; Emerson et al. 1996). In comparison with the other fingers, the thumb also has the largest cortical representation, both in the primary motor and somatosensory cortices, reflecting its extensive motor and sensory innervation (Penfield and Boldrey 1937; Janko et al. 2022). Thumb impairment is often a key contributor to loss of upper extremity function after neurologic injuries such as stroke (Connell et al. 2008; Dukelow et al. 2010; Lang et al. 2013), cerebral palsy (Bleyenheuft and Gordon 2013; Brun et al. 2021), and spinal cord injury (Anderson 2004).

Despite the thumb’s functional importance, a key aspect of thumb movement control—the proprioceptive ability to sense thumb position and movement—is poorly understood. Clinical thumb proprioception assessments rely on crude methodologies like gauging presence/absence of movement or indicating direction of applied movement (Cantero-Téllez and Medina Porqueres 2021). High-resolution, sensor-based assessments of thumb proprioception are rare; in fact, we identified only two studies that have conducted such evaluations. The first one examined the effect of thumb hypermobility on thumb proprioception by taping a laser to the participant’s thumbnail, moving their thumb so the laser pointed at a target grid, and then asking the individual to reproduce the position (Fonseka et al. 2021). Directional errors were approximately 4°–7° and did not vary with sex or age. Hypermobility had a small effect on error. The second one manually positioned the five digits of the hand on a surface and asked individuals to indicate where they felt their thumb and fingers were, relative to the wrist, on a graphical virtual environment (Dandu et al. 2020). Localization errors were 3–4 cm per digit, leading the researchers to conclude that finger (and thumb) localization is “coarse and biased”, a surprising finding given the impressive dexterity of the human hand.

While proprioceptive assessment methods for the hand and upper limb have improved in recent years, particularly with the use of robotic and tablet-based devices (Block et al. 2019; Zbytniewska-Mégret et al. 2022, 2023), these have primarily focused on finger proprioception, not the thumb specifically. Furthermore, most have assessed static, rather than dynamic, localization. Both previous studies on thumb proprioception also evaluated it under static conditions, in which the thumb was held in a location and the participant was asked to estimate the location.

Here, we sought to further characterize thumb proprioceptive ability under more complex, dynamic conditions. For this purpose, we developed a novel task in which we used a robot to move the thumb in a circle and asked participants to indicate when they felt their thumb align with a point shown on a visually displayed circle. Importantly, this involves not just spatial estimation but also predicting the timing and perceived speed of the moving thumb to time the button press with the moment the thumb passes the target. The integration of proprioceptive input with visuospatial cues and temporal prediction places additional cognitive demands on the task. This is consistent with recent proposals to expand the definition of proprioception to include such high-level, integrative processes (Wali and Block 2024).

The task we studied here is related to a task used to quantify dynamic ankle position sense in aging (Verschueren et al. 2002). For that task, a robot moved the ankle and the participant had to open their hand briskly when they perceived their ankle crossing a visual target presented on a screen. As soon as the participant pushed the button, the location of the ankle was shown with a cursor on the screen. This is essentially a linear version the task studied here for the thumb.

Prior research has shown that several factors influence proprioceptive accuracy, including movement speed and amplitude. Faster movements tend to reduce the availability and integration of sensory feedback, limiting the precision of proprioceptive estimates (Todorov and Jordan 2002; Scott 2004; Goble and Brown 2009). In contrast, larger movement amplitudes are often associated with increased muscle spindle activation and a broader range of sensory input, which may enhance proprioceptive sensitivity (Proske and Gandevia 2012). To characterize dynamic thumb proprioceptive performance, we varied the speed and diameter of movement, hypothesizing that localization would be better for larger, slower circles. We also hypothesized that thumb proprioception ability would be correlated with, but superior to, finger proprioception ability, due to its unique anatomical structure and dense sensory innervation, its essential role in dexterous hand function, and its disproportionately large cortical representation.

Finally, returning to the findings of Dandu et al. (2020), the authors demonstrated that when visual information about the index finger’s position was provided, participants’ localization of the thumb improved markedly, with error reduced by nearly half. This indicates that proprioceptive estimates can be improved using visual feedback. Aiming to understand the adaptive nature of thumb proprioception estimates, our task incorporated a propriovisual perturbation: a rotational shift between the actual thumb position and its visual representation on the screen, which was used to determine both target collisions and feedback location. We hypothesized that, similar to the Dandu et al. study, participants would adapt to this perturbation based on visual feedback of errors.

## Methodology

### Design of a novel thumb proprioception assessment

We identified three main design criteria for developing a dynamic thumb proprioception assessment task. First, we desired a task that provided a quantitative and continuous measure of thumb localization ability during movement. Second, we desired that the task be engaging. Quantitative proprioceptive testing typically requires participants to maintain extended periods of attention as they are asked to make judgements about applied movements. Varying levels of attention can confound measures of true localization error. Therefore, we embedded the assessment within an engaging videogame framework to try to minimize attention variation. Evidence supports this approach, as gamified cognitive tasks have been shown to sustain attention and motivation while maintaining scientific validity (Lumsden et al. 2016). Third, we desired that the test be applicable in the future to people with thumb motor impairment. Thus, we focused on designing a passive test that did not require active thumb movement ability and relied on a simple and comfortable imposed movement of the thumb.

**Fig. 1:**
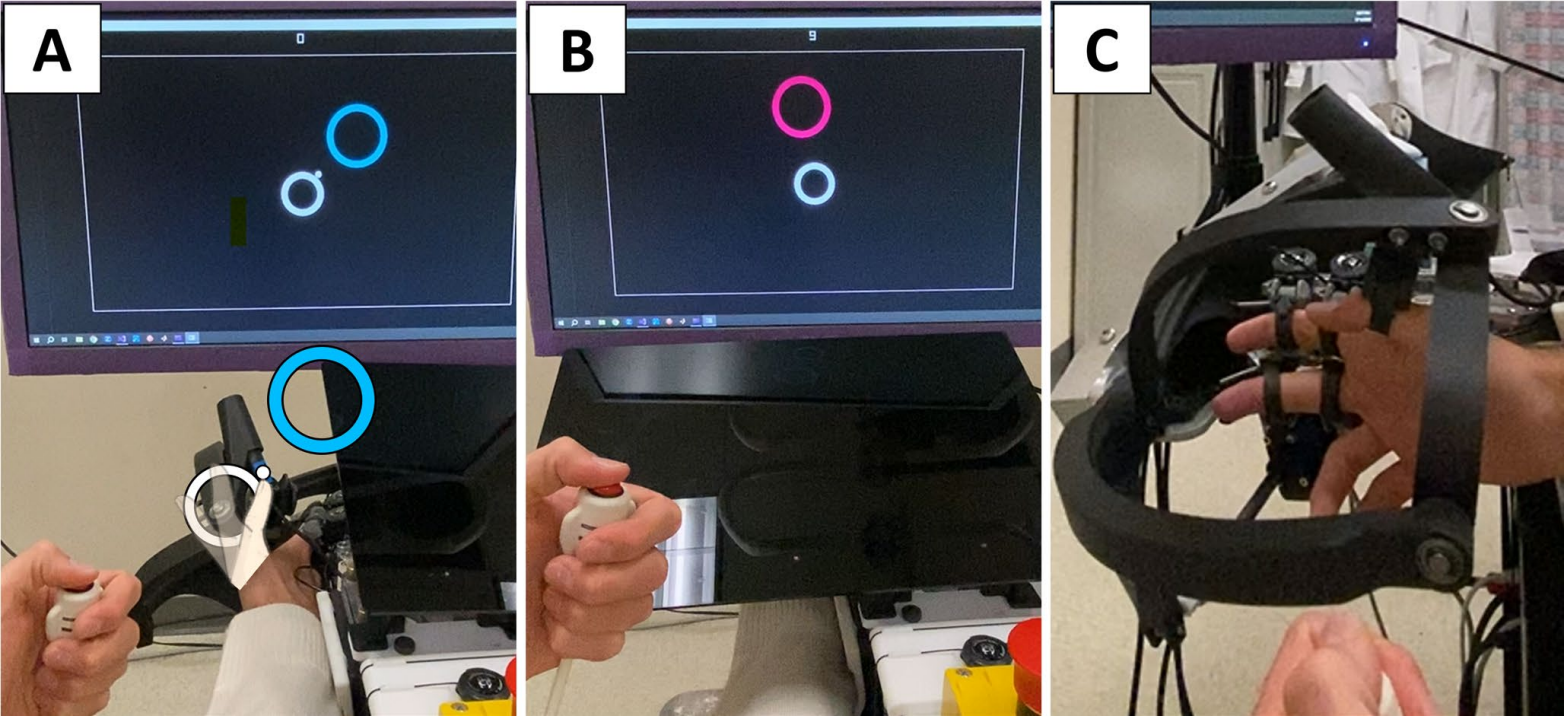
**A** Participant view of the SomatoCircleJump game with graphics overlaid in hand space to illustrate game play. The robot continuously guides the thumb in a circular path (orbiting a ~ 5 cm diameter circle in thumb space, shown by the overlaid white circle), and the participant must press a button with their other hand when the thumb is closest to the blue target circle. After each press, the target circle shifts to a new position. During task familiarization, a white cursor represents the thumb’s position and the participant has an unobstructed view of their hand. The colors of the target circles are selected from the “colorblind safe” color palette by IBM Design. **B** Participant’s point of view while performing the proprioceptive assessment. The visual aid on the screen representing the thumb’s position is removed and vision of the hand is blocked with a movable screen. **C** Side view of thumb exoskeleton of the THINGER robot used for the proprioception assessment with participant’s thumb, index, and middle fingers attached to the device. A spherical mechanism controlled the thumb in two degrees of freedom

Given these design criteria, we created SomatoCircleJump, adapted from a game known as Super Circle Jump. Super Circle Jump was originally designed for Android phones by *pixelclash* and involves the player triggering a jump when a ball, rotating around a circle, aligns with a visual target, represented by another circle (Fig. 1A). This game can also be viewed as shooting an orbiting ball at a target. For SomatoCircleJump, we modified the game by removing the visual display of the ball and instead displayed its location proprioceptively by using a robot to move the player’s thumb in a circumduction movement in a vertical plane parallel to the screen to represent the ball’s rotation around the circle. The player must press a button with their other hand to make the ball jump to the target, relying on sensing their thumb location to determine when to press the button. Although the task requires a motor output from the contralateral hand to press the button, the timing of this action must be determined by proprioceptive sensing of the passive thumb’s position relative to a visual target. No visual information about the thumb’s ongoing movement is available, so performance necessarily depends on proprioception. This interface is an example of a “Propriopixels” gaming strategy, in which a game element is presented proprioceptively rather than visually (Reinsdorf et al. 2021; Reinsdorf 2022). To play SomatoCircleJump purely proprioceptively, display of the orbiting ball is removed and vision of the thumb is occluded by a plastic screen (Fig. 1B).

The game code was based on C++ code developed previously for a finger proprioception game called Proprioceptive Pong (Reinsdorf et al. 2021). Thumb movement was applied using the THumb INdividuating Grasp Exercise Robot (THINGER) (Ketkar et al. 2023), which uses a 2-degree-of-freedom (2-DOF) spherical 5-bar thumb exoskeleton (Fig. 1C) actuated by two high bandwidth, low-friction, linear, electric actuators. THINGER is an add-on module of the Finger INdividuation Grasp Exercise Robot (FINGER) (Taheri et al. 2014).

We quantified thumb localization ability by calculating the “jump error” between the angle of the ball when the button was pressed and the optimal jump angle, which was determined by the line connecting the centers of the orbit and the target circles (Fig. 1A). We defined jump error as negative when the button was pressed before the thumb reached the target (early) and positive when pressed after the thumb passed the target (late). Participants had limited time to press the button for each target (9 s for the fast speed task, 18 s for the medium speed tasks, and 36 s for the slow speed task), with a maximum of three full rotations before the current target disappeared and the next one appeared.

**Fig. 2:**
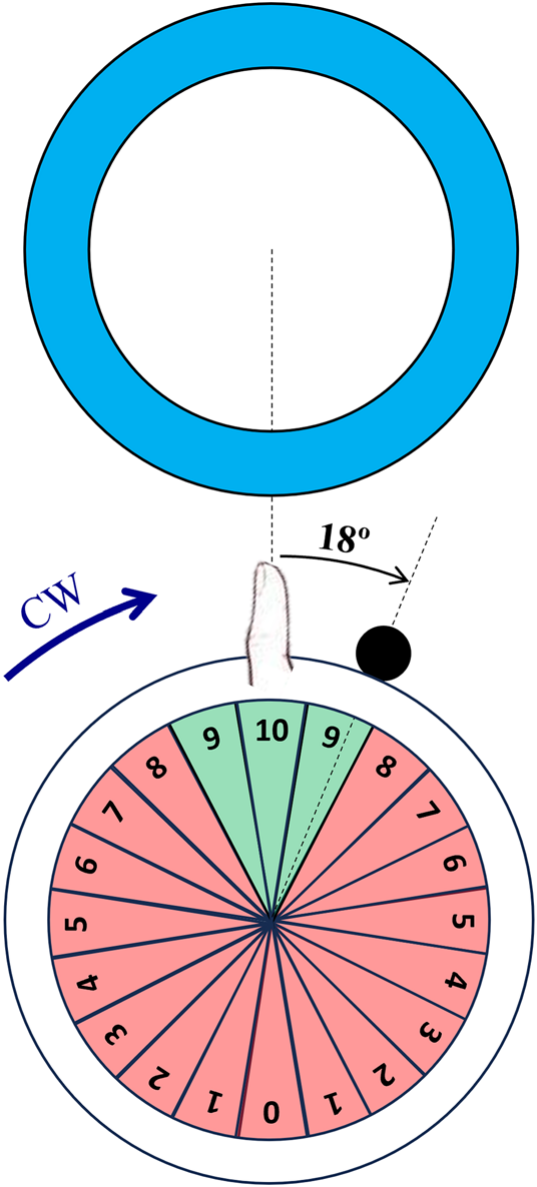
Representation of the propriovisual rotation and the feedback scoring method. For a participant playing in the clockwise (CW) direction and trying to hit a target at the top of the screen, pressing the button when their thumb was at the top position resulted in visual feedback indicating they were late, i.e. had already rotated past the target. The labeled sectors show the score values for pushing the button when the ball was in that sector. Green areas (scores 9 and 10) were considered successful

Following the button press, the SomatoCircleJump game displayed a small ball that jumped radially outward from the instantaneous location of the ball on its orbit at button press. The ball turned green if it was situated within the angular aperture that would result in a collision with the target circle (a sector subtending 54° with respect to the center of the thumb orbit), denoting a successful shot and red if it was situated outside of that angular aperture (Fig. 2). Moreover, participants received a real-time score between 0 and 10 for each jump depending on how close they were to hitting the target circle. Successful jumps were rewarded with a score of 9 (slightly early or slightly late) or 10 (right at the center of the target) (Fig. 2).

$$ \left\{ {\begin{array}{*{20}c} {\theta_{target} - 27^\circ < \theta_{jump} < \theta_{target} - 9^\circ \to Score: 9} \\ {\theta_{target} - 9^\circ < \theta_{jump} < \theta_{target} + 9^\circ \to Score: 10} \\ {\theta_{target} + 9^\circ < \theta_{jump} < \theta_{target} + 27^\circ \to Score: 9} \\ \end{array} } \right. $$

Jumps occurring outside of this sector were considered misses, with jumps occurring in each subsequent 18° sectors scored sequentially lower, such that jumps occurring between 171° and 189° from the target received a score of 0.

To probe the adaptive capability of the proprioceptive system, the game incorporated a propriovisual perturbation, implemented as an 18° clockwise rotational shift between the actual thumb location and the displayed thumb location used to determine target hits and provide feedback of ball location. With this propriovisual distortion, if the thumb was in the vertical (top) position during a clockwise rotation and the button was pressed exactly at that moment, the visual feedback would indicate that the ball was 18° to the right, causing the jump to appear to have occurred 18° late (Fig. 2). Conversely, if the rotation was counterclockwise, and the button was pressed at the same instance the visual feedback would indicate that the jump occurred 18° early, prior to the target. The value of 18° for the perturbation was arbitrarily selected and was subtle enough that no participants reported noticing the rotation. The propriovisual distortion was applied for all tasks.

### Experimental design

The Institutional Review Board of UC Irvine approved the protocol, and participants provided informed consent. Twenty-six individuals aged between 18 and 40 (23.6 ± 3.9 years old, 14 males and 12 females, 25 right-handed and one left-handed, assessed using the Edinburgh Handedness Inventory) participated. All participants had no history of neurological or finger injuries.

Participants performed the SomatoCircleJump task seven times in a single session under different experimental conditions, with each task comprising 20 jumps. We selected 20 trials per condition based on prior work using passive proprioception tasks (Ingemanson et al. 2016, 2019; Rowe et al. 2017), which has shown this number to be effective for balancing measurement reliability with participant engagement, helping to minimize the risk of reduced concentration over time. The first task was used to familiarize individuals with the game and was played with vision of both the thumb and the white ball, to allow participants to learn the virtual representation of their thumb movement on the monitor. In the subsequent six tasks (Table 1), vision of the thumb was obscured with a black screen, and no ball was shown until after participants pushed the button to indicate a jump. SomatoCircleJump experimental conditions included tasks with different thumb rotation speeds (slow ∼ 30°/s, medium ∼ 60°/s, or fast ∼ 120°/s), directions (clockwise—CW, or counterclockwise—CCW), workspace size (full or half, where full was ~ 5 cm in diameter), and employed finger (thumb or index) (Table 1). The speeds (30°/s, 60°/s, and 120°/s) were selected to provide a broad range of velocities while keeping the task feasible and engaging. The slowest speed was chosen to avoid excessively long trials, and the higher speeds were set by successive doubling to systematically sample across the range while remaining manageable in pilot testing. The full workspace tasks involved a circular motion with a diameter of ~ 5 cm, while the half workspace task used a smaller circular motion with a diameter of ~ 2.5 cm. Both involved full 360° rotations; the distinction lies in the radius of the path, not in the angular range. For the task involving the index finger, the hand was removed from its original position (Fig. 1C), and the index finger was placed in the cuff normally used for the thumb on the THINGER robot, allowing it to be rotated around its metacarpophalangeal (MCP) joint. Comparing performance in the first familiarization condition (which did not require proprioception) with the following six main tasks allowed separation of proprioceptive versus motor contributions of pressing the button to jump errors. Half of the participants conducted all tasks with a clockwise (CW) rotation as their main direction, while the other half experienced a counterclockwise (CCW) rotation.

**Table 1:** Game Parameters for each experimental task. Each task was comprised of 20 jumps

Task ID	Speed	Direction	Workspace	Finger
Standard	**Medium**	Main	Full	Thumb
Slow	**Slow**	Main	Full	Thumb
Fast	**Fast**	Main	Full	Thumb
Opposite	Medium	**Opposite**	Full	Thumb
Half	Medium	Main	**Half**	Thumb
Index	Medium	Main	Full	**Index**

In each column, the bolded entry indicates the parameter that differs from the Standard condition and defines the task ID (all non-bolded parameters are held constant)

For all participants, the initial familiarization task was performed at the medium speed in the main direction for their group, consistent with the Standard task. For the first 15 participants, the order of the subsequent six tasks was randomized. After a preliminary data analysis for these 15 participants, we observed a substantial increase in jump error when switching between movement directions, which occurred both in the Opposite task and whichever task occurred afterwards. These results appeared consistent with previously studied patterns of motor adaptation to perturbations and subsequent after effects. Thus, to ensure that after effects occurring after the Opposite task did not mask other effects of interest in subsequent tasks (i.e. speed), we decided to place the Opposite task at the end of the session for the remaining 11 participants. This further ensured that these eleven participants experienced the Opposite task at the same temporal point in the protocol, i.e. after having experienced the same amount of training in all the other five tasks (which were still randomized).

Each participant also completed Crisscross, a finger proprioception test, during the same session using the FINGER robot. In Crisscross (Ingemanson et al. 2016), the FINGER robot guides the participant’s index and middle fingers in a crossing motion, and they are prompted to press a button with their other hand at the moment they feel their fingers cross without relying on vision. Participants performed 60 total crossings at different speeds (ranging form 4°/s to 80°/s), and localization error was quantified as mean absolute crossing error across all crossings. We have shown previously that Crisscross is sensitive to aging (Ingemanson et al. 2016) and presence of stroke (Ingemanson et al. 2019), and predicts the ability to benefit from robotic hand movement training after stroke (Rowe et al. 2017).

### Data analysis

For each task, we quantified three complimentary measures of jump error following established motor-behavior methodology (Schmidt and Lee 2011). Constant error (CE), defined as the mean signed difference between the target angle and the actual angle of the ball (which represented the thumb’s position at the time of button press), captures systematic bias, with negative values indicating early presses and positive values indicating late presses. Variable error (VE), defined as the standard deviation of the signed errors relative to each subject’s own mean, quantifies within-subject consistency or precision. Absolute error (AE), defined as the mean absolute difference between the target and actual angles, combines both systematic and variable components into an overall measure of accuracy. Reporting CE, VE, and AE together provides a more complete characterization of performance, and this approach has been applied in robotic proprioception assessments (Rinderknecht et al. 2016; Zbytniewska-Mégret et al. 2023).

We identified participants who were outliers as those whose average AE across all tasks exceeded three standard deviations from the group mean. This resulted in the exclusion of the data of one of the participants from the analysis. We also conducted an outlier analysis on the trials for each task for each group, removing trials that exceeded three standard deviations from the mean for that task. Because proprioceptive tasks require sustained attention, such unusually large errors are likely to result from brief attentional lapses rather than true proprioceptive performance. This resulted in excluding 41 of the total 3120 trials (< 1.4%). Removing these rare trials did not change the overall statistical conclusions.

We used a repeated-measures ANOVA to analyze the effect of all six task conditions (Standard, Slow, Fast, Opposite, Half, and Index) on AE within a single model, allowing us to control for multiple comparisons and account for the within-subjects nature of the data. This approach enabled us to assess overall differences in performance across all task types while appropriately handling the non-independence of repeated measurements from the same participants.

To examine whether the randomization structure (i.e., whether participants experienced all tasks in random order or the Opposite task always last) influenced performance, we included randomization structure as a between-subjects factor in the repeated-measures ANOVA. This allowed us to test for main effects and interactions involving randomization structure within the same statistical framework. As there was no significant effect of randomization structure, we concluded that task order did not account for the observed effects. Therefore, data from both randomization groups were combined for all subsequent analyses.

We performed correlation analyses between thumb localization AE obtained using SomatoCircleJump and index localization AE obtained both using SomatoCircleJump and Crisscross. We checked the normality of those datasets by visually inspecting them using a histogram and a Q–Q plot, and then performed a Shapiro–Wilk test (Razali and Wah 2011). Since the data were non-normally distributed, we used Spearman’s rank correlation.

## Results

We asked participants to estimate when their thumb was aligned to radially located, visually displayed targets as we moved their thumb in a circle with a robotic device. We quantified thumb localization ability by “jump error”, defined as the difference between participants’ actual thumb angle and the cued target angle when they pushed the button (with their other hand) to indicate alignment. After the button press, participants received visual feedback of their jump CE. Throughout the task there was a rotational offset between actual thumb location and the visual feedback of thumb location presented on the screen.

### Effect of task parameters

Following familiarization with SomatoCircleJump, localization performance was consistent for the various task conditions, except the Opposite condition, suggesting that individuals had rapidly adapted to the perturbation. On average across all six tasks outlined in Table 1, mean CE was − 5.9° ± 10.4°, which was significantly less than 0 (paired t-test, *p* = 0.008), indicating a small early bias in button presses. Mean VE across tasks was 25.2° ± 5.1° and mean AE was 29.2° ± 6.2°. Task-specific CE, VE, and AE values are summarized in Table 2.

**Table 2:** Jump errors for each experimental task

Task ID	Constant error (CE) (Mean ± SD)	Variable error (VE) (Mean ± SD)	Absolute error (AE) (Mean ± SD)
Standard	− 3.0° ± 20.2°	25.2° ± 9.0°	26.2° ± 8.1°
Slow	− 14.3° ± 25.5°	25.3° ± 9.0°	29.8° ± 15.9°
Fast	− 6.5° ± 17.7°	24.5° ± 6.5°	23.7° ± 8.3°
Opposite	− 7.6° ± 48.9°	26.7° ± 7.5°	47.5° ± 14.5°
Half	− 1.4° ± 19.5°	25.9° ± 7.9°	26.0° ± 7.4°
Index	− 2.7° ± 15.8°	23.6° ± 5.3°	21.9° ± 5.7°
**Mean**	**− 5.9° ± 10.4°**	**25.2° ± 5.1°**	**29.2° ± 6.2°**

**Fig. 3:**
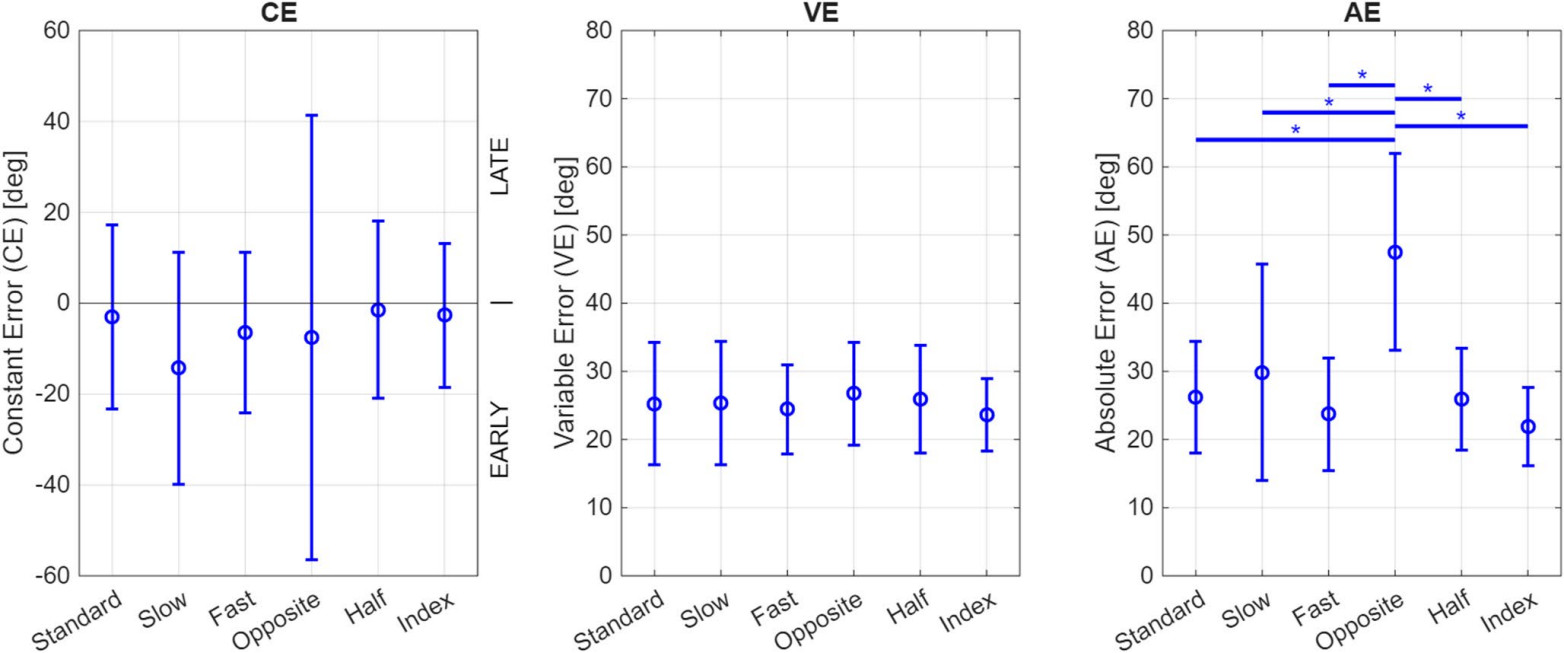
Jump constant error (CE), variable error (VE), and absolute error (AE) for the different tasks outlined in Table 1. Error bars show ± SD. Asterisks indicate pairwise comparisons with *p* < 0.001 (Bonferroni-corrected)

A repeated-measures ANOVA on the AE data, which represents the overall magnitude of the jump error regardless of direction, revealed a significant effect of Task (F(5, 120) = 26.90, *p* < 0.001, Cohen’s f = 0.82), and no significant effect of Randomization Structure (F(1, 24) = 0.16, *p* = 0.69). Post-hoc pairwise comparisons using the Bonferroni correction revealed that the Opposite direction task had significantly higher AE than all the other tasks (Fig. 3, Right, all *p* < 0.001). No significant difference was found between the other tasks. This indicated that participants had rapidly adapted to the perturbation and that error did not vary significantly with speed, workspace size, or finger used, contradicting our hypothesis that thumb proprioception would be superior to finger proprioception. In fact, index finger localization was even slightly better than thumb localization, although this difference was not statistically significant.

**Fig. 4:**
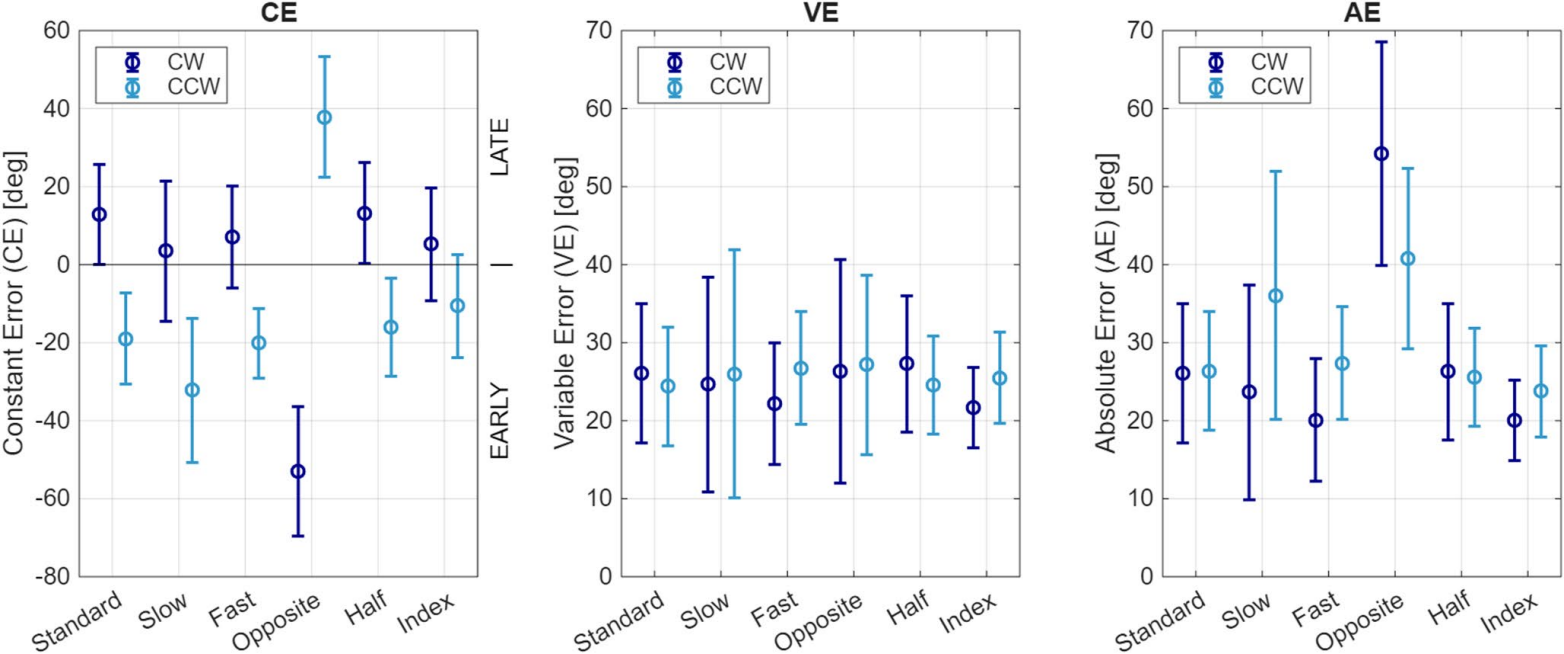
Jump constant error (CE), variable error (VE), and absolute error (AE) for the different tasks outlined in Table 1, plotted separately for the groups that primarily experienced CW rotation (dark blue) and CCW direction (light blue). Error bars show ± SD

When we changed the direction of rotation, the AE increased substantially (Fig. 3, Right). Participants in the CW group began pressing the button earlier after switching to CCW, while those in the CCW group started pressing the button later after changing to CW (Fig. 4, Left). Since the change in direction substantially increased error, we recalculated the mean AE excluding the Opposite task and found performance errors to be 25.5° ± 7.0° (significantly lower, paired t-test, *p* < 0.001). As performance showed differential timing effects between groups, we also recalculated the mean CE in each rotation direction excluding the Opposite task and found the CE to be 8.4° ± 4.5° in the CW rotation (late bias, paired t-test, *p* = 0.01), and − 19.6° ± 7.9° in the CCW direction (early bias, paired t-test, *p* = 0.005).

No improvement in overall localization accuracy was observed across the six experimental tasks totaling 120 jumps, as there was no significant difference in AE between the first and last task (paired t-test, *p* = 0.96). The Opposite direction condition was excluded from this analysis because participants who completed it as their final task had not yet fully adapted to the change in rotation direction, resulting in elevated errors on their 120th jump.

### Trial-by-trial adaptation

**Fig. 5:**
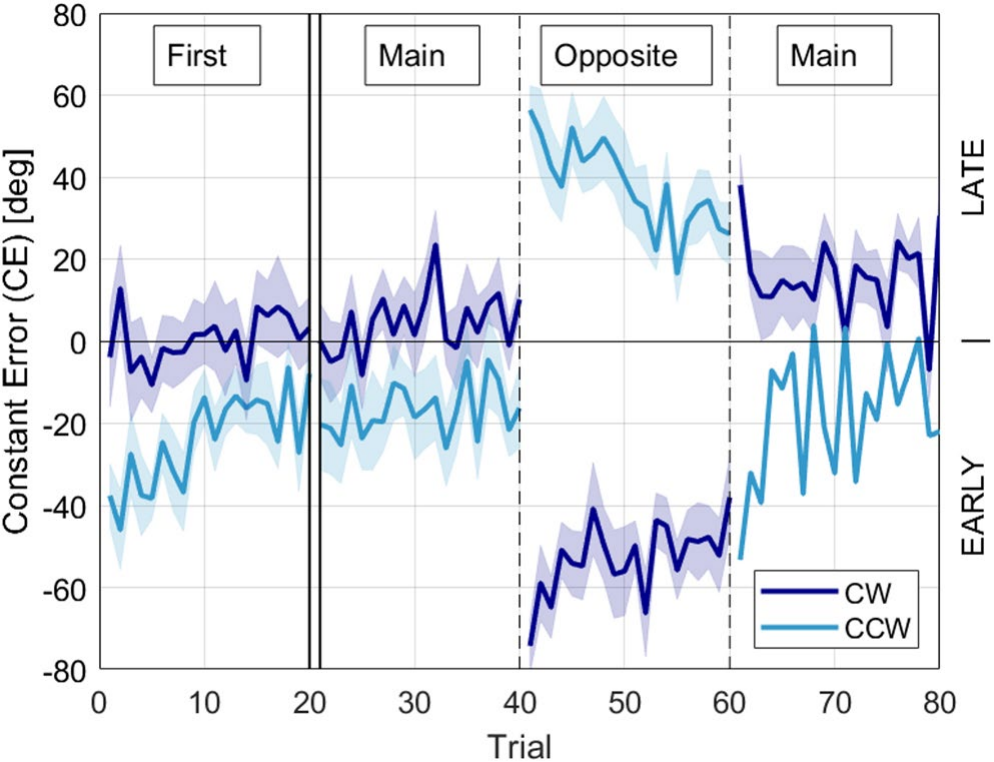
Mean constant error (CE) on the first game, and before, during, and after changing directions for groups whose main directions were CW (dark blue) and CCW (light blue). The double vertical lines indicate a temporal gap, as some participants completed other tasks between their initial game and the task immediately preceding the direction change. The dashed lines represent divisions between consecutive tasks. Shaded regions show ± SE

To characterize the trial-by-trial reaction to introduction of the perturbation and the changing rotation direction, we plotted the average jump CE for all participants for their first task and the tasks right before, during, and after the Opposite task (Fig. 5). During the first task, CE decreased over the first 10 trials, indicating rapid adaptation to the perturbation. Following the initial increase in CE magnitude due to the switch in rotation direction, the magnitude of CE quickly decreased over the next 20 trials (Fig. 5, trials 40–60). This adaptation pattern was reminiscent of error patterns during motor adaptation experiments with robotic force fields or visuomotor rotations (Shadmehr and Mussa-Ivaldishad 1994; Della-Maggiore et al. 2015) and we will adopt the terminology used in those experiments to describe effects.

Specifically, when we switched rotation direction from trial 40 to 41, the magnitude of CE more than doubled (paired t-test 40th to 41st trial, *p* < 0.001), similar to a “direct effect” of applying a perturbation in adaptation paradigms. The direction of the direct effect depended on the initial rotation direction (Fig. 5, trial 40, cf. CW to CCW). Subsequently, the amplitude of the error decreased over the next 20 jumps for both CW and CCW groups, indicating significant adaptation (paired t-test 41st to 60th trial, *p* < 0.001). At trial 61, when we reverted the rotation direction to the original direction, there was an “after effect”, the direction of which again depended on the rotation direction (Fig. 5).

This pattern can be understood better by closely examining initial error patterns prior to the change in rotation direction, and considering the design of the propriovisual distortion. For the first trial of the first game, the jump CE was − 4.0° ± 12.0° for the CW rotation group (i.e. slightly early) and − 37.6° ± 7.8° for the CCW rotation direction (i.e. very early) (Fig. 5). This disparate first trial error between groups was caused by the propriovisual rotation between the position of the thumb and the ball on the screen, and by the fact that all participants, regardless of the direction of rotation, on average initially pushed the button slightly early, prior to physical alignment with the target; in the CW case, the effect of the propriovisual perturbation (18° clockwise rotational visual shift) would then reduce the effect of the anticipatory error. In contrast, if people similarly pushed the button too early in the CCW direction, the propriovisual perturbation would further increase the error.

**Fig. 6:**
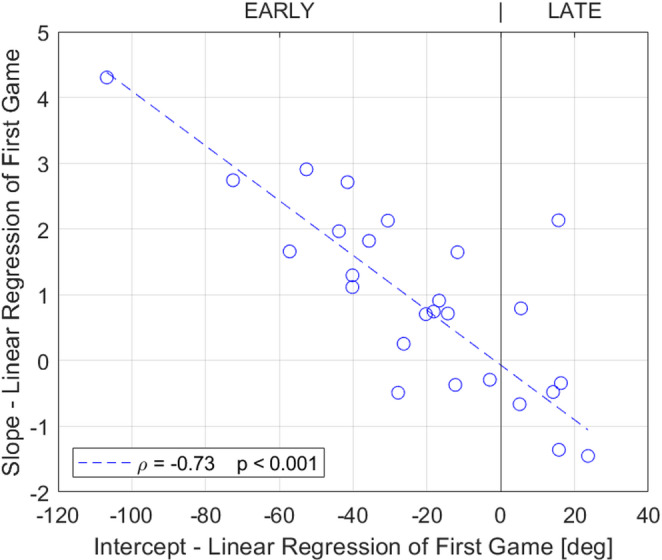
Correlation analysis of the slope and intercept parameters from the linear regressions of each participant’s first SomatoCircleJump game. Dashed line shows robust fit, and statistics show results of correlation using Spearman’s Rank correlation. A high positive initial error (intercept) correlated with a negative slope, indicating adaptation to the propriovisual rotation. Conversely, a high negative initial error correlated with a positive slope, while participants with a small initial error exhibited a slope close to zero, reflecting minimal adaptation

For the CCW group, this large, initial error of the first game provoked adaptation across the next 20 jumps (Fig. 5). Thus, by the 20th jump, the error for this group was significantly less than the 1st trial (paired t-test 1st to 20th trial, *p* = 0.04), because they pushed the button later. In contrast, the CW group did not adapt (paired t-test 1st to 20th trial, *p* = 0.5), and we hypothesized that this was because their error was relatively small. To test this hypothesis, we plotted the amount of adaptation versus the initial CE in the first exposure and found that they were correlated (Fig. 6, ρ = − 0.73, *p* < 0.001). If their initial error was small, participants did not adjust. If their initial error was large, they gradually pushed the button either earlier or later to reduce error, based on the direction of the initial error indicated by the visual feedback after each jump. That is, initial adaptation was error driven. This allowed individuals to reduce error compared to their initial performance, where errors were spread across a wide range, due in part to the perturbation but also reflecting substantial variability beyond the error expected due to the perturbation.

**Fig. 7:**
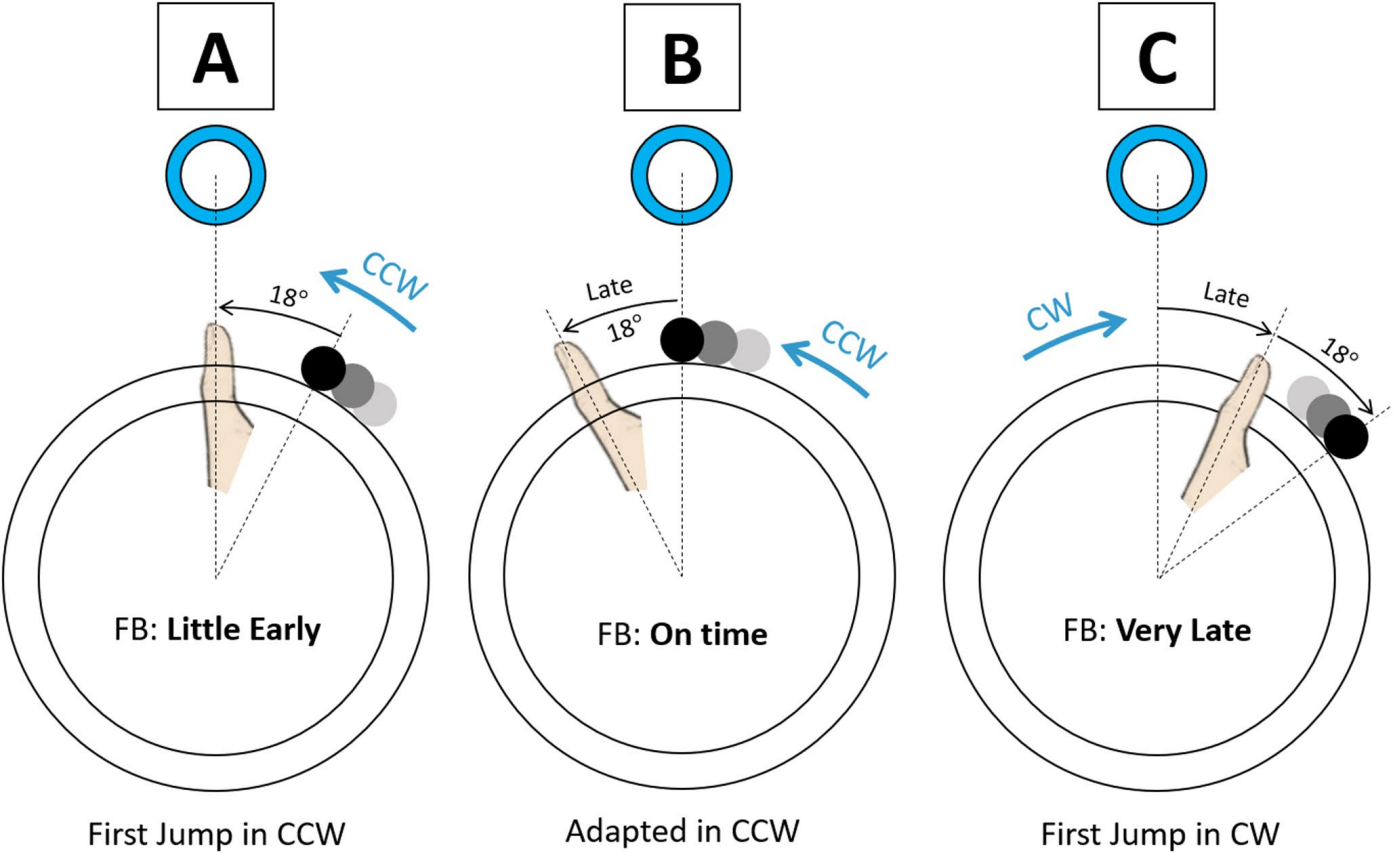
Illustration of body-centered frame adaptation in the group that initially played with counterclockwise (CCW) rotation with hypothetical top position targets. **A** When the propriovisual perturbation was introduced, pressing the button when the thumb was actually aligned with the target resulted in feedback (FB) indicating an early press, before the visually rotated ball had reached the target. **B** To reduce this error, participants gradually adapted by pressing the button later, after their thumb had passed the target. **C** In the Opposite task, where the rotation direction switched to clockwise (CW), this adapted strategy led to overshooting, with late presses now resulting in feedback indicating the response was too late

Returning now to the moment the rotation direction reversed (trial 40 in Fig. 5), the error changed in opposite directions for the groups that experienced the CW or CCW rotations initially. For the CW group that had grown used to pressing the button before their thumb reached the actual target position, when the rotation was abruptly changed to CCW their predisposition to pressing the button early added to the 18° CW propriovisual perturbation made them be very early. Conversely, the CCW group had learned to push the button later following their initial exposure to the task. When the rotation direction was abruptly changed to CW while maintaining the same 18° CW rotation, pressing the button late now resulted in being very late, since the CW rotation now added to the effect of their lateness (Fig. 7). Thus, the earlier adaptation pattern based on a body-centered reference frame helps explain the large, opposite responses to the direction change.

### Correlation between thumb and finger localization error

**Fig. 8:**
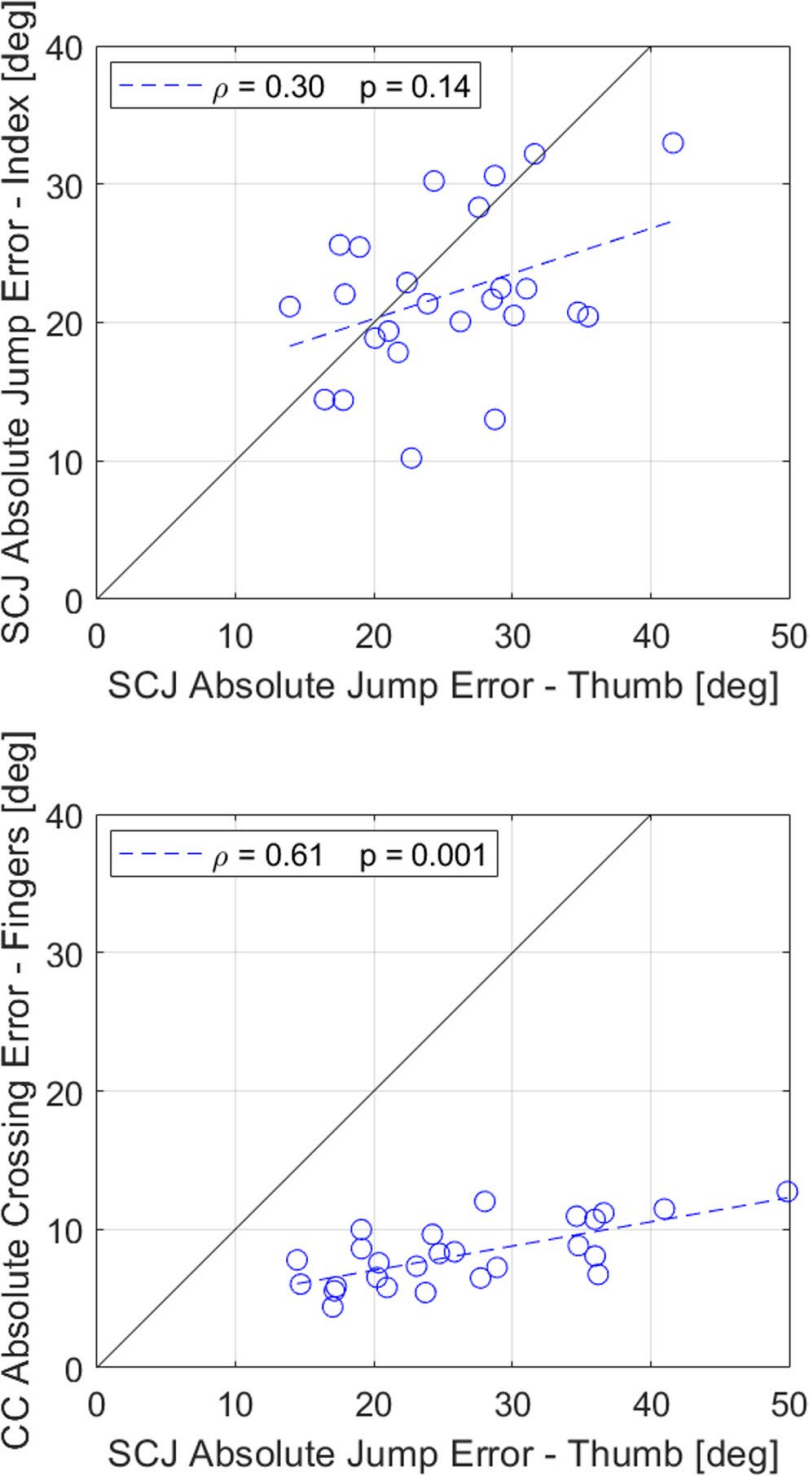
Correlation analyses between thumb proprioception jump absolute error (AE) and finger proprioception AE. Black solid lines show slope of 1, blue dashed lines show robust fit, and statistics show results of correlation using Spearman’s Rank correlation. Top: Thumb vs Index finger jump AE from SomatoCircleJump (SCJ). Bottom: Thumb jump AE from SomatoCircleJump vs Index and Middle fingers crossing AE from Crisscross (CC)

Earlier we reported that AE was similar when playing with the index finger and with the thumb (Table 2). To determine if thumb and index finger errors were correlated, we plotted the AE obtained in the Standard task against the AE obtained in the Index task and found that they were not significantly correlated (Fig. 8, Top, ρ = 0.30, *p* = 0.14).

However, we also compared thumb jump AE with finger proprioception error measured with the Crisscross test. Since the participants performed the Crisscross task at different speeds, the thumb’s mean AE across SomatoCircleJump’s Standard, Slow, and Fast tasks was calculated for each participant and compared to their index and middle fingers’ mean crossing AE in the Crisscross test. Thumb and finger errors were significantly correlated for these different testing approaches (Fig. 8, Bottom, ρ = 0.61, *p* = 0.001). Thumb jump errors were systematically larger than finger crossing errors, as can be seen from the regression line.

### Decomposing localization error

In this experiment we measured the ability of individuals to predict when their thumb would pass a target using their thumb proprioceptive sense. This task required individuals to push a button to indicate their estimate of thumb position. The motor action of pushing the button has associated with it some variability, as every motor action has. The variability in pushing the button would, in this task, show up as increased localization error.

To quantify the contribution of inherent button pressing variability to thumb localization error, we analyzed data from a warm-up condition in which individuals performed the SomatoCircleJump task but with a cursor shown on the screen that showed the exact location of the thumb as it moved. In this case there is no ambiguity about when to push the button: the participant can clearly see when the thumb cursor passes the target. Across participants, the mean CE in the motor-only condition was -2.4°, with a VE of 6.9°. In comparison, the Standard condition under otherwise identical conditions but without cursor vision (in which case localization error includes both proprioceptive and motor contributions) yielded mean CE and VE values of 13.0° and 26.1° for the CW group, and -19.0° and 24.4° for the CCW group (Fig. 4, Left and Center, Standard task). These comparisons suggest that approximately 18.5% of the bias and 26.4% of the variability for the CW group, and 12.6% of the bias and 28.3% of the variability for the CCW group, could be attributed specifically to motor variability in the button press.

## Discussion

Thumb proprioception is poorly characterized at present. One of the few initial studies found that it is surprisingly coarse and biased, but only measured static conditions (Dandu et al. 2020). For further characterization, we developed a novel, dynamic thumb localization assessment in which participants relied on their thumb position sense to discern the position of an orbiting ball to be able to shoot it at targets. Thumb localization error during this dynamic task had a magnitude of ~ 25° on average, with a smaller mean bias of ~ 6° “early”, showing that thumb localization is coarse also in dynamic conditions. When participants performed the familiarization trials with vision of the thumb cursor on the screen, the localization error, which arose only from errors in motor timing since participants knew exactly where the thumb was from the visual cursor, was much smaller. This indicates that the larger localization errors in the main tasks primarily reflect proprioceptive rather than motor contributions. However, thumb proprioception demonstrated two notable proficiencies as we varied task parameters. First, localization error was largely unaffected by rotation speed and diameter, indicating a degree of robustness to these parameters. Second, participants quickly improved their performance using visual feedback of errors. Finally, we hypothesized that thumb proprioception would be correlated with, but superior to, finger proprioception. The two were not significantly correlated for SomatoCircleJump, and finger proprioception showed a trend toward being superior, but the difference was not statistically significant. Thumb jump error and finger Crisscross error were significantly correlated, with, again, finger error being smaller. We now discuss these findings and suggest directions for future research to continue to improve the understanding of thumb proprioception.

### Dynamic characteristics of thumb proprioception

There have been few studies of thumb proprioceptive ability, but one recent study manually placed the fingers and thumb in different configurations and asked participants to indicate the perceived positions of their fingertips without visual feedback, using a visual cursor moved with a mouse in a virtual reality environment (Dandu et al. 2020). Using this static positioning methodology, they found an average error of 3.7 cm per digit with significant biases. This led the investigators to conclude that finger localization is coarse and biased. They also showed that the localization of the thumb and index finger was the most accurate of the digits (mean error of 2.9 cm and 2.8 cm respectively). In the present study, we found an average localization error of ~ 25°, which corresponds to an error of approximately 1.1 cm for thumb localization, still coarse but less than half the error in this previous study.

Two possible explanations for why dynamic thumb localization was more accurate than the static localization in Dandu et al. (2020) are as follows. First, some evidence suggests that proprioceptive localization improves when it is frequently visually recalibrated. For instance, in the Dandu et al. (2020) study they also performed an experiment where they showed the position of the index finger in the virtual reality environment and asked the participants to locate their thumb. This indirect visual information significantly reduced the localization error to 1.5 cm (48% reduction). In the present study, adaptation would be expected to have occurred through the visual display of the thumb’s position on the screen every time the participants pressed the button to initiate a jump. And, indeed, as we discuss below, the observation of rapid adaptation to the propriovisual rotational perturbation supports the idea of visual feedback aiding proprioceptive accuracy.

A second possibility is that the thumb proprioceptive system leverages velocity-related information to estimate thumb position. Muscle spindles are highly sensitive to velocity and integrating a high-fidelity velocity signal could, in theory, improve position estimates, consistent with sensor fusion algorithms in robotic sensing (Grill and Hallett 1995; Macefield and Knellwolf 2018; Alatise and Hancke 2020). Thus, it may be that the dynamic nature of the task reduced localization errors. In this case, however, one might have expected error to depend on rotation speed, which it did not, as we discuss below.

It is worth noting that most of the thumb proprioception error we measured was due to variability rather than systematic bias, since the CE was only ~ 5°. This pattern was reflected in the decomposition of errors into CE, VE, and AE: AE represents the overall magnitude of error (~ 25°), while VE contributed the majority of this error and CE was relatively small, consistent with a low but statistically significant early bias. Pushing the button early is consistent with the idea that the proprioceptive system overestimated the thumb speed at the speeds we tested. Nevertheless, the larger contribution to the thumb proprioceptive AE in the current study was the error variability from trial to trial, reflecting “proprioceptive uncertainty”.

In another recent study on thumb proprioception, researchers assessed joint position reproduction (JPR) by attaching a laser to the participant’s thumbnail. They guided the thumb so that the laser pointed at a target grid and then asked participants to replicate the position. The study reported localization errors ranging from 4° to 7° (Fonseka et al. 2021), which correspond approximately to a localization error of 0.3 cm on the tip of the thumb. The localization error observed in the SomatoCircleJump task was larger, which might be attributed to key differences in task design. In JPR tasks, participants have the advantage of experiencing and memorizing the target positions before attempting to reproduce them, possibly contributing to improved accuracy. In contrast, SomatoCircleJump requires participants to detect target positions in real-time without prior exposure, potentially making it more challenging to refine their internal representation of the target location. Additionally, the range of motion in SomatoCircleJump was considerably larger (360° vs. 120°) and more complex (circumduction movement vs. flexion/extension movement) than in the previously mentioned JPR task, which may contribute to increased error due to greater spatial uncertainty over a wider movement trajectory.

Given the large errors we measured, an unexpected finding was that varying the rotation speed or diameter did not significantly affect error. The SomatoCircleJump task is a dynamic task that requires anticipating when the thumb will reach the target position; faster or smaller amounts of thumb movement might be expected to degrade estimates. However, this was not the case. Interestingly, while not statistically significant, we observed a trend toward higher errors and greater variability at the slower speed condition, opposite to our original hypothesis. We can think of several possible explanations for the lack of dependence of error on rotation speed and diameter.

First, the lack of significant change in thumb localization error with varying speeds might be attributable to the observation that muscle spindles increase their firing rates at higher speeds, maintaining sensitivity to rapid changes in muscle length and ensuring that proprioceptive feedback remains reliable (Macefield and Knellwolf 2018). Additionally, the CNS integrates the information provided by the spindles with other sensory inputs (e.g., joint receptors, cutaneous feedback) and predictive internal models to estimate limb position. These internal models, refined through experience, allow the CNS to maintain accurate localization even during faster movements (Proske and Gandevia 2012; Li et al. 2023). Furthermore, the task design, which provided visual error feedback after each jump, facilitated error correction, possibly also reducing the impact of speed on localization accuracy. Thus, the combined contributions of improved muscle spindle sensitivity at higher speeds, CNS integration of velocity-sensitive models, and error feedback may help explain why thumb localization error remained consistent across speeds. Taken together, these findings may reflect a generalization of proprioceptive acuity across different task conditions, supported by both sensory mechanisms and adaptive feedback. That said, it is also possible that the range of speeds used in this study was not sufficiently broad to elicit robust differences in performance. Future work using a wider range of movement velocities and perhaps limited feedback could help address these possibilities.

The possible reasons why reducing the workspace size did not increase error are less clear to us. Halving the size of the circular motion reduced in half the thumb localization error in the world coordinate frame to ~ 0.55 cm. Skin stretch, a contributor to joint position sense (Gandevia et al. 1983), would be expected to be less for the smaller circles, but that would then be expected to increase error. Perhaps the somatosensory system uses the recent history of experienced range of motion to adjust proprioceptive sensitivity dynamically, increasing it for smaller movement sequences. Alternatively, feedback may have facilitated recalibration of proprioceptive mappings, allowing participants to maintain high levels of performance. This is an interesting direction for future research.

### Adaptability of thumb proprioception to a propriovisual perturbation

Initial error was large, due in part to the perturbation but also because of a large amount of thumb localization uncertainty at the beginning of the task (see Fig. 6). However, we found that thumb localization ability improved quickly with visual error feedback. This does not mean that the task became a visually driven task: while feedback allowed participants to improve their performance, these improvements necessarily depended on proprioceptive information. Specifically, participants were informed whether they pressed the button too early or too late, but such information is useless on its own. Without proprioceptive input about thumb position, their performance would remain at chance. Thus, any error-based adjustments must be integrated with proprioceptive information about thumb location. Therefore, we maintain that the task fundamentally quantifies proprioceptive ability even though it provided visual error feedback, with feedback serving to help the sensory motor system adjust its response and not replacing reliance on the underlying proprioceptive signal.

There are two possible mechanisms by which this adaptive response could occur. First, the proprioceptive system could have recalibrated based on the visual error feedback, such that it perceived the thumb at a different location than it was. Alternately, the motor system could have learned to adjust the button push response to the perceived thumb location by updating the internal model between thumb position and motor response. Both proprioceptive recalibration and internal model updating could occur simultaneously; future research should determine their relative contributions.

Of note, our findings suggest that, for either mechanism, the adaptation was not based on learning a fixed angular offset in a world reference frame (e.g., always adjusting 18° CW regardless of movement direction), but rather on a relative adjustment defined in the frame of reference aligned with the thumb’s movement. Specifically, participants appeared to adapt by applying a fixed angular shift either forward or backward along their movement direction, with forward shifts when initially moving CW and backward shifts when initially moving CCW. This implies that adaptation occurred in a reference frame anchored to the thumb’s motion direction, rather than one fixed in the external world. This is consistent with findings that proprioception is typically represented in egocentric rather than allocentric reference frames (Blouin et al. 1993; Moraresku and Vlcek 2020). In contrast, a world-based model would involve always applying the same angular correction (e.g., 18° CW) regardless of movement direction.

This interpretation is supported by the asymmetric error patterns observed when the direction of rotation was reversed (Fig. 4). If participants had learned to compensate using a world-based model (e.g., always correcting 18° CW), switching the direction of rotation should not have substantially affected their accuracy, as the angular discrepancy would remain constant. Instead, the significant increase in error at the start of the Opposite task, and the direction-specific differences depending on whether participants began with CW or CCW rotation, strongly suggest a movement-frame-based strategy. In this model, the correction (either proprioceptive or motor) is not applied uniformly in a fixed spatial direction, but rather in the direction aligned with the original thumb movement, whether that is forward or backward. Determining when the sensory motor system elects to use a world-reference frame versus a relative frame of reference for internal model formation is an interesting direction for future research.

The adaptation we observed was a form of implicit learning, referring to the automatic acquisition of new movement patterns through experience without explicit awareness or intention. While the baseline SomatoCircleJump is consistent with an explicit learning task because participants are consciously aware of the instruction to press a button when they perceive their thumb to reach the target, we use the term “implicit” to refer to the adaptation because participants were not consciously aware of the mismatch between their thumb position and the visual cue; at no point did we inform them about the presence of the propriovisual rotation, and none of the participants spontaneously reported noticing this manipulation. Instead, they adapted to the rotation by gradually adjusting their responses to minimize the error, without explicit instruction or conscious recognition of the perturbation. Furthermore, the presence of after effects when the rotation direction was switched supports the interpretation that the adaptation was implicit, since an explicit strategy would have reversed immediately without producing such after effects. Implicit learning of a proprioceptive-visual mismatch during passive arm movement has been observed before (Cressman and Henriques 2010; Salomonczyk et al. 2013). Specifically, exposure to propriovisual discrepancies in the absence of voluntary movement leads to rapid sensory recalibration. The discrepancy between seen and felt positions has been termed the “cross-sensory error signal” and has been hypothesized to drive changes in perceived hand position (Salomonczyk et al. 2013). The adaptation we observed here could potential involve cross-sensory error adaptation, although we cannot rule out there was a form of motor adaptation as well.

### Comparison between thumb and finger proprioception

Counter to our expectations, using the index finger instead of the thumb in the SomatoCircleJump test reduced jump error, although not significantly. We expected the thumb to have better proprioception because of its larger representations in somatosensory cortex (Martuzzi et al. 2014; Janko et al. 2022). One possibility is that the better proprioceptive ability of the index finger can be attributed to an aspect of the task studied here: i.e. the way the digit cuff of the THINGER robot pulled on the thumb versus finger. Some participants reported feeling a greater amount of skin stretch when they put their index finger in the robot versus the thumb. This additional tactile feedback may have enhanced their ability to estimate the position of their index finger and could also have disrupted a true correlation between thumb and index finger SomatoCircleJump performance. Counter to this explanation, however, was the finding that thumb proprioception was not significantly better for larger diameter rotations, which presumably stretched the skin around the thumb more than the smaller diameter rotations.

Despite the similar (and slightly better) localization ability of the index finger, the proprioceptive ability of the fingers (measured with Crisscross) and thumb (measured with SomatoCircleJump) were moderately correlated. It is well established that proprioceptive ability is modifiable with exercise and training (Aman et al. 2015; Winter et al. 2022). Thus, the correlation between finger and thumb proprioceptive ability may be due to the fact that the index and middle fingers and the thumb are used together in many activities. People who are more active with their hands would be expected to have better finger and thumb proprioception. Conducting a study in which finger or thumb proprioception are trained together or selectively would help test this possibility.

Although finger localization error in Crisscross was correlated with thumb localization error in SomatoCircleJump, the finger error was significantly smaller. This difference may be due to the fact that Crisscross involved judging relative positions between digits, which is likely easier than the absolute spatial localization required in SomatoCircleJump. The presence of a propriovisual distortion in SomatoCircleJump may have further increased localization error. These differences make the correlation between the two tasks particularly surprising, given their distinct demands.

### Future directions to improve understanding of thumb proprioception

The results of this study contribute new knowledge regarding dynamic aspects of thumb proprioception, but many questions remain. We outline a few interesting future directions for research here.

It was surprising that rotation speed and diameter did not affect localization accuracy. Exploring a greater range of speeds and diameters could provide further insight into the limits of this robustness. Using cutaneous anesthesia could help identify the role of skin stretch in thumb proprioception. The finding of a rapid, movement-dependent adaptation to the propriovisual rotation raises several interesting questions, such as the extent the adaptation depends on the perturbation magnitude, what factors determine whether individuals form a body-centered versus world-centered internal model, and the relative roles of proprioceptive and motor recalibration. Removing visual feedback on some trials would provide insight into the error dynamics of recalibration. Adaptation in response to visual feedback is likely essential to thumb dexterity, given the high proprioceptive sensing variability of the thumb, and should be further examined to more fully understand hand dexterity. Additionally, future studies could include bilateral assessments to evaluate the influence of handedness on thumb proprioception, providing insight into lateralization effects and informing individualized approaches to thumb sensorimotor rehabilitation.

We developed the experimental protocol described here to be suitable for people with thumb movement impairments. We have already applied the SomatoCircleJump assessment to stroke survivors to investigate proprioceptive decline resulting from neurological injuries (Garcia-Fernandez et al. 2025). Thumb proprioception after stroke is rarely evaluated in the clinic (Hillier et al. 2015; Valdes and Rider 2024), and when it is, it is typically evaluated using qualitative methods, such as determining the presence or absence of movement and indicating the direction of applied movement as assessed by a clinician (Cantero-Téllez and Medina Porqueres 2021). The novel robotic and gamified assessment we developed here could potentially improve this situation, particularly if the assessment could be implemented on simpler mechatronic devices. Along these lines, we recently developed a 3D-printed robot with hobby-grade components that can implement SomatoCircleJump (Garcia-Fernandez and Reinkensmeyer 2025).

A recent systematic review of nearly 1350 methods for assessing proprioception asserted there is little relationship among the measures obtained by the different testing procedures, emphasizing the joint-specificity and method-specificity of proprioceptive testing (Han et al. 2013; Horváth et al. 2023). The moderate correlation we found between thumb and index/middle finger proprioception errors using two distinct tasks (SomatoCircleJump and Crisscross) challenges these findings, suggesting that it is possible to find generalizable aspects of proprioceptive ability in unimpaired adults, at least for the hand.

Lastly, while this study focused on thumb proprioception, the same paradigm could be adapted to assess other joints with at least two degrees of freedom (such as the wrist, elbow, shoulder, neck, hip, and ankle). Additionally, we aim to investigate the potential of SomatoCircleJump as a proprioception training tool, leveraging its engaging and enjoyable video game format.

## Data Availability

No datasets were generated or analysed during the current study.
